# Outpatient hysteroscopy impact on subsequent assisted reproductive technology: a systematic review and meta-analysis in patients with normal transvaginal sonography or hysterosalpingography images

**DOI:** 10.1186/s12958-024-01191-0

**Published:** 2024-02-01

**Authors:** Jung-Hsiu Hou, Buo-Jia Lu, Ya-Li Huang, Chih-Heng Chen, Chi-Huang Chen

**Affiliations:** 1 Division of Reproductive Medicine, Department of Obstetrics and Gynecology, Taipei Medical University Hospital, Taipei, Taiwan; 2 Graduate Institute of Medical Science, College of Medicine, Taipei Medical University, Taipei, Taiwan; 3 Department of Public Health, School of Medicine, College of Medicine, Taipei Medical University, Taipei, Taiwan; 4 Department of Urology, National Taiwan University Hospital, Taipei, Taiwan; 5 Department of Obstetrics and Gynecology, School of Medicine, College of Medicine, Taipei Medical University, Taipei, Taiwan

**Keywords:** Infertility, Artificial reproductive technology, Office hysteroscopy, Outpatient hysteroscopy, Diagnostic hysteroscopy, Hysterosalpingography, Transvaginal sonography, Transvaginal ultrasound

## Abstract

**Background:**

Standard management for intrauterine lesions typically involves initial imaging followed by operative hysteroscopy for suspicious findings. However, the efficacy of routine outpatient hysteroscopy in women undergoing assisted reproductive technology (ART) remains uncertain due to a lack of decisive high-quality evidence. This study aimed to determine whether outpatient hysteroscopy is beneficial for infertile women who have unremarkable imaging results prior to undergoing ART.

**Methods:**

A systematic review and meta-analysis were conducted following PRISMA guidelines, incorporating data up to May 31, 2023, from databases such as PubMed, Embase, and the Cochrane Library. The primary outcome assessed was the live birth rate, with secondary outcomes including chemical pregnancy, clinical pregnancy rates, and miscarriage rates. Statistical analysis involved calculating risk ratios with 95% confidence intervals and assessing heterogeneity with the I^2^ statistic.

**Results:**

The analysis included ten randomized control trials. Receiving outpatient hysteroscopy before undergoing ART was associated with increased live birth (RR 1.22, 95% CI 1.03–1.45, I^2^ 61%) and clinical pregnancy rate (RR 1.27 95% CI 1.10–1.47, I^2^ 53%). Miscarriage rates did not differ significantly (RR 1.25, CI 0.90–1.76, I^2^ 50%). Subgroup analyses did not show a significant difference in clinical pregnancy rates when comparing normal versus abnormal hysteroscopic findings (RR 1.01, CI 0.78–1.32, I^2^ 38%). We analyzed data using both intention-to-treat and per-protocol approaches, and our findings were consistent across both analytical methods.

**Conclusions:**

Office hysteroscopy may enhance live birth and clinical pregnancy rates in infertile women undergoing ART, even when previous imaging studies show no apparent intrauterine lesions. Treating lesions not detected by imaging may improve ART outcomes. The most commonly missed lesions are endometrial polyps, submucosal fibroids and endometritis, which are all known to affect ART success rates. The findings suggested that hysteroscopy, given its diagnostic accuracy and patient tolerability, should be considered in the management of infertility.

**Database registration:**

The study was registered in the International Prospective Register of Systemic Review database (CRD42023476403).

**Supplementary Information:**

The online version contains supplementary material available at 10.1186/s12958-024-01191-0.

## Background

Recent global estimates suggest that roughly one in six individuals of reproductive age may encounter infertility during their lifetime [1]. In females, the causes of infertility are categorized into ovulatory, tubal/uterine, other, and unexplained [2]. Uterine cavity abnormalities are present in approximately 10% of subfertile women, and nearly half of those with recurrent implantation failure may have abnormal uterine conditions [3]. Therefore, evaluating the uterine cavity is a routine part of the initial infertility assessment in women.

Uterine factors, including endometrial polyps, fibroids, intrauterine synechiae, and congenital malformations, may distort the uterine cavity [4]. Common methods for assessing the uterine cavity in subfertile women include transvaginal sonography (TVS), saline infusion sonohysterography, and hysterosalpingography (HSG). Advanced imaging techniques such as 3D ultrasound or MRI are also options, particularly for conditions such as bicornuate uterus. More invasive procedures, such as hysteroscopy or laparoscopy, are also used [3, 5]. Hysteroscopy, being the gold standard, not only allows direct observation of lesions but also enables pathology confirmation through biopsy and potential immediate surgical correction. As such, operative hysteroscopy, which combines diagnosis and treatment, is the preferred method when suspicious lesions are detected in imaging studies.

Outpatient or office hysteroscopy has gained popularity due to advancements in endoscopic technology, which include smaller hysteroscopes and enhanced visual systems, making the procedure more feasible and acceptable [6]. Its main advantage is the avoidance of general anesthesia, reducing related risks and costs, and improving patient acceptance. Despite a reduction in instrument size, office hysteroscopes often retain a working channel, allowing biopsy or removal of small lesions. This has led more physicians to adopt outpatient hysteroscopy as a routine infertility evaluation. Nevertheless, studies have shown that outpatient hysteroscopy, saline infusion sonography, and hysterosalpingography have comparable diagnostic accuracy for uterine cavity assessment in infertile women [7]. Since outpatient hysteroscopy is more invasive and lacks evidence that it improves pregnancy rates, current guidelines recommend its use only when clinically indicated, rather than as a first-step evaluation [8, 9].

To date, compelling high-quality evidence supporting the routine use of hysteroscopy as a diagnostic tool in the evaluation of infertility—particularly in women poised for assisted reproductive technology with unremarkable transvaginal ultrasound or hysterosalpingography results—is lacking. Given the increase in assisted reproductive treatment cycles and the growing adoption of outpatient hysteroscopy, it is imperative to re-evaluate its role as an additional diagnostic tool within the infertile population. This study aims to determine whether there is a definitive benefit to routinely incorporating hysteroscopy into the diagnostic process for infertility, especially for patients with negative imaging studies who are candidates for assisted reproductive treatment.

## Methods

### Search methods and eligibility criteria

We performed a comprehensive search of electronic databases, including PubMed, Embase, and the Cochrane Library, up to May 31, 2023, to identify pertinent studies. Our search strategy adhered to the Preferred Reporting Items for Systematic Reviews and Meta-Analyses (PRISMA) guidelines. We employed the following search terms: ICSI (intracytoplasmic sperm injection), IVF (in vitro fertilization), fertilization, transfer, infertility, ART (assisted reproductive technology), reproductive technolog*, in combination with “Hysteroscop*” and “resectoscop*”.

Our analysis was confined to randomized controlled trials (RCTs) to ensure a high level of evidence and to minimize potential bias. We established predefined inclusion criteria for patient populations, radiological assessment, and control groups. Studies that satisfied all three of these criteria were considered for inclusion:

- *Patient population*: We included infertile women slated for further assisted reproductive technology procedures, including patients either undergoing their first cycle of in vitro fertilization (IVF) or those with a history of previous IVF failure. We excluded patients pursing natural conception, undergoing dating only or those receiving intrauterine insemination (IUI).
- *Radiological assessment*: Eligible studies were needed to have confirmed the absence of gross uterine abnormalities through normal transvaginal sonography (TVS) or hysterosalpingography (HSG) prior to the study.
- *Control group*: Studies must have a control group that did not undergo hysteroscopy as part of their diagnostic or treatment regimen. These patients proceeded directly to further assisted reproductive technology (ART) without an outpatient hysteroscopy examination.

The primary outcome we focused on was the live birth rate, with secondary outcomes encompassing the chemical pregnancy rate, clinical pregnancy rate, and miscarriage rate. These outcomes were chosen to provide a comprehensive assessment of the reproductive results following hysteroscopy in women undergoing ART.

### Study selection and data extraction

Two reviewers (Hou and Lu) independently screened titles and abstracts for relevance. Full texts of potentially eligible studies were then examined based on our criteria. Any disagreements were resolved through discussion or, if necessary, by consulting a third reviewer (Chen).

Data from the selected RCTs were extracted using a standardized form by two independent reviewers. Extracted information included study characteristics, participant demographics, intervention details, and relevant outcomes. Additionally, abnormal findings and subsequent management of hysteroscopy were recorded. In-depth details regarding infertility-associated features, ART protocols, and the hysteroscopy process are provided as supplementary data.

### Appraisal of methodological quality

Three reviewers (Hou, Lu, and Huang) assessed the methodological quality of the included RCTs using the Cochrane Risk of Bias tool for randomized controlled trials 2.0 [10]. This tool evaluates biases across multiple domains such as random sequence generation, allocation concealment, blinding, incomplete outcome data, selective reporting, and other biases. Studies were classified as having low, unclear, or high risk of bias in each domain. The methodological quality assessment for each study is detailed in the supplementary data (Supplementary Fig. S1).

### Statistical analysis

Data synthesis and statistical analysis were conducted in line with PRISMA guidelines [11, 12] using Review Manager version 5.3. We calculated the relative risk (RR) with 95% confidence intervals (CIs) to evaluate binary variables. A random-effects model was employed to accommodate potential study heterogeneity, which we quantified using the I^2^ statistic, classifying it as low (25–50%), moderate (50–75%) and high (> 75%) [13].

Analyses were conducted using both intention-to-treat (ITT) and per-protocol (PP) approaches. The ITT analysis included all RCT participants, while the PP analysis focused on those who completed the study and received ART, excluding any participants lost to follow-up. The primary data presentation is based on the PP approach, given our specific interest in ART pregnancy rates. ITT data was also presented and the further forest plot figure was illustrated in Supplementary Figure S2.

## Results

### Search results

**Fig. 1:**
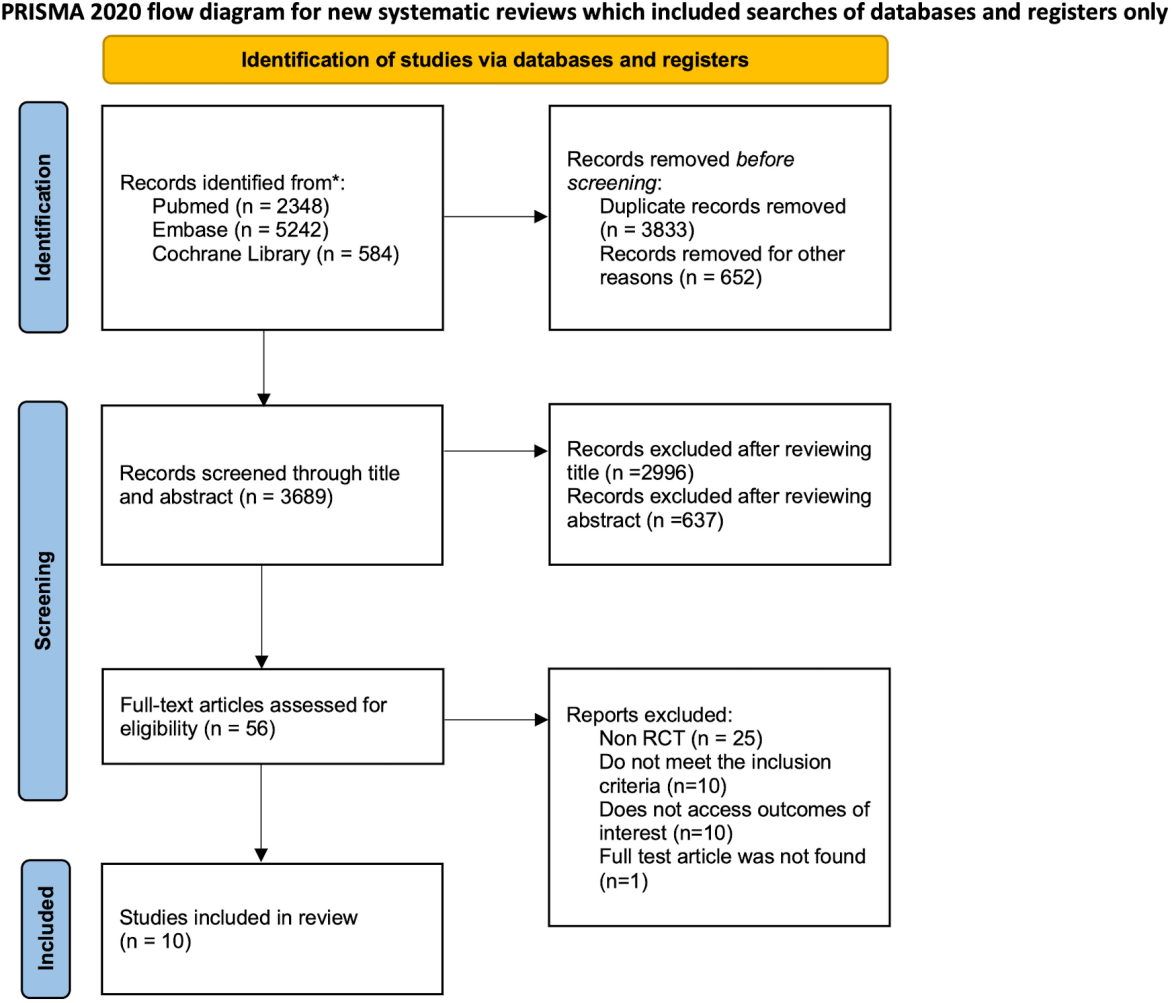
Study screening and inclusion process following the PRISMA guideline

Following the PRISMA guidelines, our systematic review process is depicted in Fig. 1. Initially, 3689 records were identified for further screening. After reviewing titles and abstracts, 56 articles were assessed for eligibility. Of these, 46 articles were excluded for various reasons: 25 were non-RCTs, 20 did not meet our inclusion criteria, such as lacking a control group design, including patients with natural pregnancy or including patients undergoing intrauterine insemination, 10 did not report the outcomes of interest, 21 were not in English, and one were inaccessible in full text. Although three articles did not provide data on the primary outcome of live birth rate, they were included in this meta-analysis due to their reporting of other relevant secondary outcomes [14–16]. Ultimately, 10 RCTs were incorporated into this meta-analysis [17–23].

### Trial characteristics

Published between 2004 and 2022, these 10 RCTs comprised 3612 patients, with 1795 receiving outpatient hysteroscopy before commencing ART and 1817 proceeding directly to ART. The salient features of the included articles are summarized in Table 1. Study settings varied: seven were single-center, one spanned two centers, and two were multicenter across Europe. Patient ages ranged from 27 to 33 years. Concerning fertilization methods, two study solely used IVF, three used ICSI exclusively, and five tailored the use of IVF or ICSI to the clinical situation. Regarding embryo transfer methods, seven studies utilized fresh embryo transfer, two reported frozen embryo transfer, and one did not document this information. Detailed characteristics of the infertility conditions and treatment protocols are presented in supplementary table S1.

**Table 1:** Characteristics of the included randomized control trials

Author, publication year	Study area	Main inclusion criteria	Main exclusion criteria	Intervention	Control	Live birth rate (HSC/Control)	Clinical pregnancy rate (HSC/Control)	Conclusion
Demirol et al. (2004)	Single center in Turkey	≥ 2 previous implantation failure		Outpatient HSC (*N* = 210)	Immediate IVF (*N* = 211)	NA	32.1%/21.5%	HSC improves PR.
Raju et al. (2006)	Single clinic in India	≥ 2 previous implantation failure		Outpatient HSC (*N* = 265)	Immediate IVF (*N* = 255)	28.5%/16.8%	43.1%/26.3%	HSC improves LBR and PR.
Shawki et al. (2012)	Single center in Egypt	For first or further ICSI		Outpatient HSC (*N* = 105)	Immediate IVF (*N* = 110)	37.8%/28.0%	44.4%/30.0%	HSC improves PR but does not improve LBR.
Elsetohy et al. (2015)	Single center in Egypt	For first ICSI	Recurrent miscarriage	Outpatient HSC (*N* = 97)	Immediate IVF (*N* = 96)	59.8%/34.4%	70.1%/45.8%	HSC improves LBR and PR.
Alleyassin et al. (2015)	Single center in Iran	For first ICSI	Recurrent miscarriage	Outpatient HSC (*N* = 110)	Immediate IVF (*N* = 110)	NA	48.2%/38.2%	HSC improves PR.
Smit et al. (2016)	Multicenter in Netherlands	For first IVF or ICSI	≥ 2 miscarriages	Outpatient HSC (*N* = 369)	Immediate IVF (*N* = 373)	54.5%/52.3%	NA	HSC does not improve LBR.
El-Toukhy et al. (2016)	Multicenter in UK, Belgium, Italy, Czech Republic	Age < 38 y/o 2–4 times previous implantation failure	BMI > 35 kg/m² ≥ 37 y/o with < 8 oocytes retrieved previously	Outpatient HSC (*N* = 350)	Immediate IVF (*N* = 352)	31.6%/33.1%	37.9%/37.9%	HSC does not improve LBR or PR.
Abid et al. (2021)	Single center in Tunisia	Age < 40 y/o BMI ≤ 30 kg/m² For first IVF		Outpatient HSC (*N* = 84)	Immediate IVF (*N* = 87)	25.0%/19.3%	32.4%/28.9%	HSC does not improve LBR or PR.
Pounikar et al. (2022)	Two centers in India	Age < 45 y/o ≥ 1 previous implantation failure	First time IVF	Outpatient HSC (*N* = 90)	Immediate IVF (*N* = 90)	NA	30.0%/23.3%	HSC improves PR.
Ghasemi et al. (2022)	Single center in Iran	Age ≤ 40 year For first IVF	≥ 3 miscarriage	Outpatient HSC and irritation of uterine cavity (*N* = 109)	Immediate IVF (*N* = 119)	57.8%/47.9%	63.3%/58.0%	HSC improves cumulative LBR.

HSC, hysteroscopy; PR, clinical pregnancy rate; LBR, live birth rate; ICSI, intracytoplasmic sperm injection; IVF, in vitro fertilization

### Quality of included studies

Four studies were assessed as having low risk of bias, while five exhibited some concerns, primarily due to insufficient details about the randomization process and when patients were informed of their allocation. Blinding is challenging in invasive procedures such as hysteroscopy; however, one study was deemed at high risk of bias due to a significant disparity in the number of patients with only one prior IVF failure between the hysteroscopy and control groups. Such disparities could influence the live birth rate, our primary outcome of interest.

### Primary outcome

**Fig. 2:**
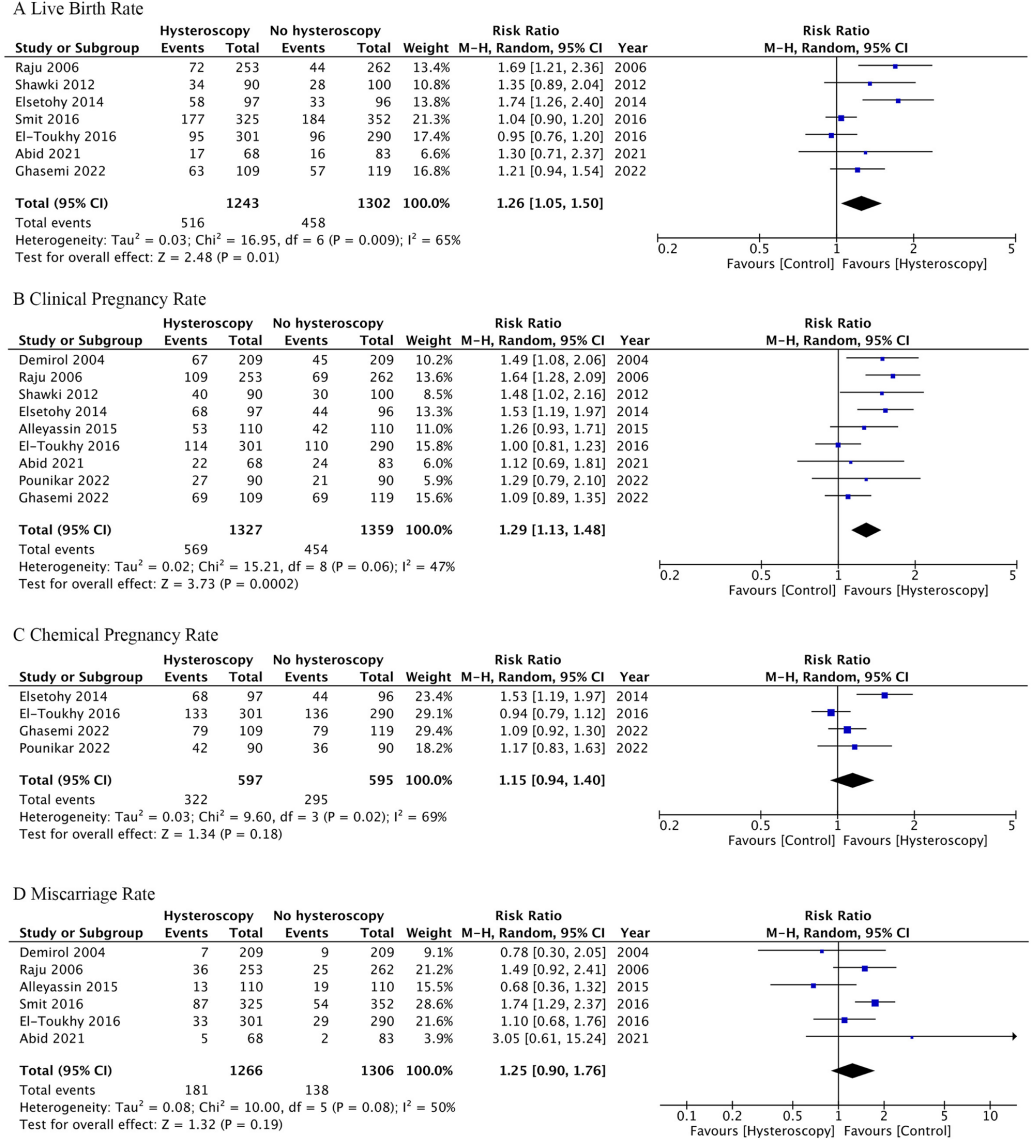
Forest plot comparing outcomes between the hysteroscopy and control groups. There was a significant increase in (**A**) live birth rate and (**B**) clinical pregnancy rate among the outpatient hysteroscopy group. There was no significant difference in (**C**) chemical pregnancy rate or (**D**) miscarriage rate between the two groups. These results were analyzed through per-protocol approach. The results analyzed through intention-to-treat approach was showed in Fig. S2

Seven studies reported live birth rates, defined as the delivery of a viable baby after 24 weeks of gestation. For patients with prior negative transvaginal ultrasound or hysterosalpingography, outpatient hysteroscopy was associated with a significant increase in live birth rates (RR 1.26, CI 1.05–1.50, *I*^*2*^ 65%, analyzed by the per-protocol approach; RR 1.22, CI 1.03–1.45, *I*^*2*^ 61%, analyzed by the intention-to-treat approach) (Fig. 2A and Fig. S2A). The degree of heterogeneity was moderate (I^2^ = 65% and 61%, by per-protocol and intention-to-treat approach, respectively).

### Secondary outcome

Clinical pregnancy rates were documented in nine studies, with clinical pregnancy defined as the visualization of fetal heartbeat via ultrasound. The hysteroscopy group demonstrated a significantly higher clinical pregnancy rate than the control group (RR 1.29, CI 1.13–1.48, *I*^*2*^ 47%, analyzed by the per-protocol approach; RR 1.27, CI 1.10–1.47, *I*^*2*^ 53%, analyzed by the intention-to-treat approach) (Fig. 2B and Fig. S2B), indicating low heterogeneity.

Four studies examined chemical pregnancy rates, typically defined as a positive beta-human chorionic gonadotropin blood test 14 days post-embryo transfer. There was no significant difference in chemical pregnancy rates between the groups (RR 1.15, CI 0.94–1.40, *I*^*2*^ 69%, analyzed by the per-protocol approach; RR 1.13, CI 0.92–1.38, *I*^*2*^ 68%, analyzed by the intention-to-treat approach) (Fig. 2C and Fig S2C), with moderate heterogeneity observed. Similarly, six studies reported miscarriage rates, finding no significant difference between the groups (RR 1.25, CI 0.90–1.76, *I*^*2*^ 50%, analyzed by the per-protocol approach; RR 1.25, CI 0.93–1.69, *I*^*2*^ 38%, analyzed by the intention-to-treat approach) (Fig. 2D and Fig S2D), accompanied by low heterogeneity.

### Subgroup analysis according to the hysteroscopic findings

**Fig. 3:**
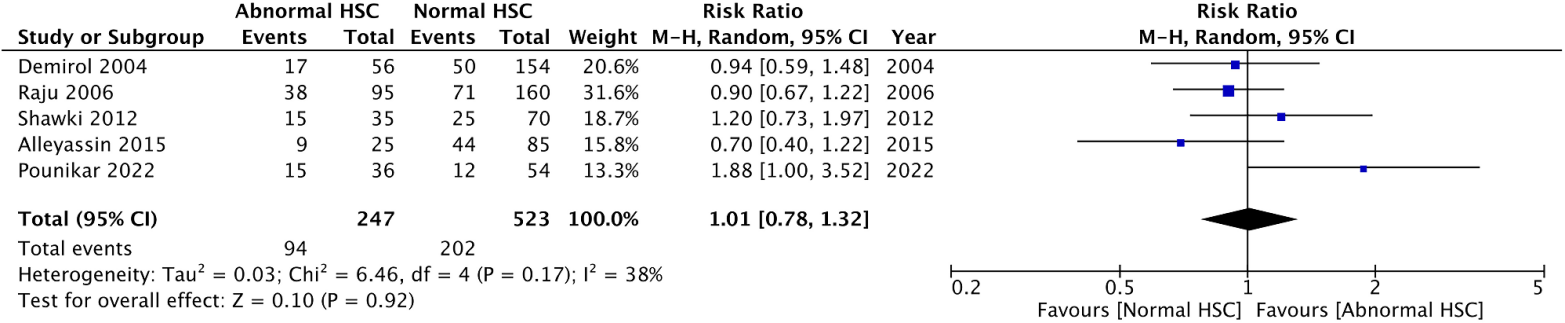
Forest plot depicting subgroup analysis of clinical pregnancy rates among the hysteroscopy group. Comparing outcomes between the normal hysteroscopic finding and abnormal hysteroscopic finding groups, there was no significant difference in the clinical pregnancy rate

A subgroup analysis within the hysteroscopy cohort explored the impact of normal versus abnormal findings on ART outcomes. Five studies compared clinical pregnancy rates between patients with normal and abnormal findings. Common practice among these studies was to biopsy and treat any detected abnormalities. The analysis revealed no significant difference in clinical pregnancy rates based on hysteroscopy findings (RR 1.01, CI 0.78–1.32, *I*^*2*^ 38%) (Fig. 3).

### Abnormal hysteroscopy findings

Nine studies provided details on the diagnosis of abnormal hysteroscopic findings. These lesions, not identified by transvaginal sonography or hysterosalpingography, were detected through outpatient hysteroscopy. The studies indicated a false-negative rate for imaging studies between 13% and 43%. The most commonly identified new lesions via outpatient hysteroscopy included endometrial polyps, found in 3–15.7% of cases; intrauterine adhesions, in 1–13%; submucosal fibroids, in 0.4–7.2%; endometritis, in 1.9–8.3%; septate or arcuate uterus, in 0.9–13.6%; and endometrial hyperplasia, in 1.8–4.8%. Additionally, cervical adhesion or stenosis was noted in 2.3–12.0% of procedures. The comprehensive findings from hysteroscopy are presented in Table 2.

**Table 2:** Characteristics and findings of hysteroscopy in included RCTs

Author, publication year	Abnormal hysteroscopy	Etiology of unsuspected lesions^1^	Instrument	Anesthesia/ Sedation	Timing of procedure/subsequent ART
Demirol et al. (2004)	26.7%	EM polyps 15.7% IUA 8.5% Cervix stenosis 2.3%	5 mm diameter with 5 Fr working channel, 30° view (Bettocchi)	Midazolam 0.1 mg/kg IV when needed	Early follicular phase/NA
Raju et al. (2006)	38.0%	EM polyps 12.8% Abn Cervix 12% IUA 4.8% EM hyperplasia 4.8% Fibroids 0.4% Anomalies^2^ 3.2%	5 mm diameter with 5 Fr working channel, 30° view (Krishna)	Midazolam 0.1 mg/kg IV when needed	NA/NA
Shawki et al. (2012)	33.4%	EM polyps 10.8% IUA 3.8% Fibroids 3.8% Endometritis 1.9% Anomalies^2^ 0.9% EM hyperplasia 2.8%	3.5 mm diameter, 0° view (Versascope, Gynecare, Ethicon, Sommerville, NJ, USA)	None	NA/NA
Elsetohy et al. (2014)	43.3%	EM polyps 13.4% Anomalies^2^ 10.4% Fibroids 7.2% IUA 6.2%	4.3 mm diameter with 5 Fr working channel, 30° view	NA	Early to mid-follicular phase/Start ICSI cycle within 3 months
Alleyassin et al. (2015)	22.7%	Anomalies^2^ 13.6% EM polyps 5.4% IUA 0.9% EM hyperplasia 1.8%	4 mm diameter rigid hysteroscopy, 30°view	None	Mid-luteal phase/Start ICSI next cycle
Smit et al. (2016)	13.0%	EM polyps 9.5% Anomalies^2^ 2.3% Fibroids 1.3% IUA 1%	5 mm diameter with 5 Fr working channel, 30° view	Paracervical block when needed	Early to mid-follicular phase/Start ICSI cycle within 3 months
El-Toukhy et al. (2016)	26.0%	Anomalies^2^ 8% Cx stenosis 4% EM polyps 3%^3^ IUA 0.9%	2.9 mm diameter rigid hysteroscopy, 30° view (TROPHYscope; Karl Storz, Tuttlingen)	None	Follicular phase/Started IVF in the following month
Abid et al. (2021)	31.0%	IUA 13% EM polyps 8.3% Endometritis 8.3% Fibroids 1.2%	2.9 mm diameter (26,120 BA STORZ)	Analgesic treatment level 2 (Paracetamol/Codeine 500 mg/30 mg) 1 h before the procedure	Mid-follicular phase/Started IVF in the following month
Pounikar et al. (2022)	40.0%	NA	NA	NA	NA/Started IVF in the following month
Ghasemi et al. (2022)	NA	NA	5 Fr working channel, 30° view^4^	NA	Early to mid-follicular phase/NA

ART, artificial reproductive treatment; EM, endometrial; IUA, intrauterine adhesion; Cx, cervix; IV, intravenous; NA, not available; ICSI, intracytoplasmic sperm injection

^1^ The percentage was calculated based on the number of patients who received hysteroscopy

^2^ The anomalies include septate, arcuate, or bicornuate uterus

^3^ The incidence included 1% micropolyps

^4^ The study did not report the diameter of the hysteroscopy

## Discussion

The analysis of live birth and clinical pregnancy rates both showed a significant improvement in patients who underwent outpatient hysteroscopy compared to those who did not. This beneficial effect persisted across both intention-to-treat and per-protocol analyses. The finding that outpatient hysteroscopy can positively influence live birth and clinical pregnancy rates is promising, suggesting that the treatment of subtle intrauterine pathologies, potentially missed by conventional imaging, can substantially enhance reproductive outcomes.

Our study examined the impact of outpatient hysteroscopy, detailing the procedural specifics in Table 2. Ben Abid et al. (2021) preemptively administered level 2 analgesic treatment (paracetamol with codeine, 500 mg/30 mg) one hour before the procedure [23]. Other studies did not routinely prescribe anesthesia or sedation but provided midazolam (0.1 mg/kg IV) or a paracervical block as needed. The majority of patients allocated to the hysteroscopy group underwent the examination successfully. Smit et al. (2016) reported a procedural failure rate of 8.9%, which might be overestimated since a 5-mm outer diameter continuous flow hysteroscope was employed [17, 24]. Utilizing a 3.5-mm mini hysteroscope can reduce discomfort, enhance visualization, and increase the success rate [25]. Notably, the included studies reported no adverse events associated with outpatient hysteroscopy.

Operative findings and final diagnoses from hysteroscopy in the included studies indicated a false-negative rate between 13% and 43%. This rate of abnormal findings is in line with Karayalcin et al. (2010), who reported a 22.9% prevalence of endometrial pathology in 2,500 cases undergoing hysteroscopy before IVF [26]. Variability in incidence rates across studies can be attributed to differences in patient selection. For instance, the prevalence of uterine fibroids or endometrial polyps tends to increase with age [27, 28]. Additionally, many studies did not stratify by symptomatic indications for anomalies or differentiate patients with normal sonography.

Emerging evidence suggests that intrauterine lesions can decrease pregnancy likelihood, and their treatment may enhance pregnancy rates and ART success. Bosteels et al. (2018) assessed the effect of hysteroscopic removal of endometrial polyps, submucous fibroids, uterine septum, or intrauterine adhesions in subfertile women or those undergoing IUI, IVF, or ICSI. Despite the evidence being of low to moderate quality, their findings suggest that removing submucous fibroids and endometrial polyps can improve the chances of clinical pregnancy [29]. In our study’s subgroup analysis, the clinical pregnancy rate showed no significant difference between normal and abnormal hysteroscopic findings. Also, the physicians of the included studies would manage the abnormal lesions found through hysteroscopy as their medical routine. Due to the above reasons, we assumed that treating lesions identified by outpatient hysteroscopy might contribute to improved clinical pregnancy rates.

Hysteroscopy can detect smaller lesions and ascertain their precise location more accurately than imaging studies. The impact of lesion size on subsequent pregnancy remains unclear; however, evidence indicates that even small lesions can disrupt implantation. For instance, Perez-Medina et al. (2005) reported improved pregnancy rates following the removal of polyps smaller than 1 cm [30]. Additionally, micropolyps are now considered indicative of chronic endometritis [31], and lesion location is crucial, particularly those at the uterotubal junction [32]. Therefore, hysteroscopy may uncover small or critically located lesions that require appropriate management.

Compared to major abnormal intrauterine lesions associated with infertility, diagnosing chronic endometritis (CE) through transvaginal sonography or hysterosalpingography is challenging. CE, characterized by persistent local inflammation, can significantly impede reproductive success [33]. Often asymptomatic, CE frequently goes undetected by affected women and clinicians alike. The hallmark of CE is the presence of plasma cells within the endometrial stroma [34], necessitating histological diagnosis through hematoxylin and eosin (H&E) staining or immunohistochemistry (IHC) for Syndecan-1 (CD138) following endometrial sampling or biopsy. CD138 has proven more sensitive for diagnosing CE, with a reported prevalence of 10.4% in infertile women [35]. Additionally, hysteroscopic observation of the endometrium without biopsy serves as an alternative diagnostic method. Typical hysteroscopic hallmarks of CE include hyperemia, edema, and micropolyps [31], with a diagnostic accuracy of 93.4% when these criteria are combined. Hence, hysteroscopy, with or without biopsy, is effective for diagnosing CE, potentially enhancing the pregnancy rate among infertile patients.

Beyond detecting and treating abnormal lesions, outpatient hysteroscopy itself may have a beneficial impact on pregnancy outcomes. Instrument passage through the cervical canal allows for the early detection and resolution of cervical issues prior to ART. The fluid infusion and irrigation during hysteroscopy could mechanically cleanse the endometrium, potentially eliminating anti-adhesive glycoproteins and fostering a more implantation-conducive environment [36, 37]. Increased pregnancy and live birth rates after hysterosalpingography have been documented [38, 39], likely due to uterine flushing, which removes tubal debris and modulates macrophage secretion of interleukins and prostaglandins [40–42]. While hysteroscopy employs a gentler flushing force and utilizes fluid instead of contrast media, its pregnancy rate improvement may share a similar mechanism with HSG. Additionally, the included studies reported no major complications. The reported complication rate for diagnostic hysteroscopy was 0.13%, significantly lower than that for operative hysteroscopy. Perforation was the most commonly reported complication in diagnostic hysteroscopy [43].

The consensus on whether to perform outpatient hysteroscopy in patients without intrauterine lesions on imaging is still evolving. Two thorough systematic reviews and meta-analyses by Di Spiezio Sardo et al. (2016) and Kamath et al. (2019) reported improved live birth and clinical pregnancy rates in hysteroscopy groups but stopped short of endorsing routine screening hysteroscopy due to the quality of evidence [44, 45]. Notably, our study population and inclusion criteria differed from these analyses. Di Spiezio Sardo et al. (2016) included studies that compared “operative” hysteroscopy with the absence of the procedure, which might potentially overemphasize the benefits of hysteroscopy. Conversely, Kamath et al. (2019) included studies examining the impact of outpatient hysteroscopy on intrauterine insemination. We excluded patients undergoing IUI for two main reasons: First, our goal is to provide more precise insights for patients considering invasive ART, such as IVF or ICSI, who typically deliberate more frequently on whether to undergo various procedures than those opting for natural conception or IUI. Second, cost-efficiency plays a crucial role in deciding to proceed with a procedure. Given the significantly higher costs of IVF or ICSI compared to IUI, we believe it is justifiable to focus our study exclusively on patients undergoing IVF or ICSI. Also, our inclusion was strictly for studies confirming normal transvaginal sonography or hysterosalpingography results, which was different from other studies. Although doing so will reduce the patient population that our research can be applied to, we hope that such results can provide clearer answers to the questions of this smaller group of patients. Furthermore, we analyzed data using both intention-to-treat and per-protocol approaches. Considering that ART is elective and many factors can influence the decision to proceed with it, per-protocol analysis—based on patients who eventually received ART rather than all those included in the trial—is more reflective of real-world scenarios. Our findings were consistent across both analytical methods.

The ESHRE add-ons working group recently concluded that screening hysteroscopy does not substantially improve live birth rates when performed prior to initiating IVF treatments. However, they acknowledged the potential benefits for patients with recurrent implantation failure [8, 46]. This conclusion was primarily drawn from findings by Kamath et al. (2019), who observed improved live birth and clinical pregnancy rates in the hysteroscopy group [45]. Despite our results indicating a statistical improvement in these rates, we concur with the ESHRE add-ons working group’s cautious recommendation. The decision to perform an add-on procedure such as hysteroscopy requires a comprehensive assessment that considers not only pregnancy outcomes but also cost-effectiveness.

The prevalence of undetected intrauterine abnormalities in asymptomatic patients varies widely, with reports as low as 11% [47]. Conversely, our findings suggested that office hysteroscopy could increase the chance of live birth by 1.2 times. When combining the prevalence of undetected lesions with the relative risk improvement, hysteroscopy resulted in six additional live births per 100 procedures. This simplistic calculation offers a preliminary cost‒benefit analysis adaptable to varying IVF and hysteroscopy costs across regions. A more sophisticated cost-effectiveness evaluation was undertaken by Kasius et al. (2013), suggesting that routine hysteroscopy before IVF could be economically viable. Their model, based on data from the Netherlands in 2013, estimated the costs per IVF cycle and per screening hysteroscopy at $2726 and $134, respectively [48]. However, an updated, region-specific analysis is warranted, considering that diagnostic rates, live birth rates from IVF, and associated costs are subject to change over time and differ by location.

In our analysis, we refined the research question to focus on a more specific subset of the population than previous studies. Nonetheless, this study has limitations. The quality of the included RCTs varied, with some being small-scale and having bias concerns, including one study with a high risk of bias [14]. Sensitivity analyses, excluding the study with high risk, did not alter our results. The invasive nature of hysteroscopy precludes the possibility of double-blinded RCTs, contributing to moderate to high heterogeneity in our results. Another limitation of our study is that various patient characteristics, such as recurrent implantation failure, the cause of infertility, or the indications for choosing ICSI/IVF, could affect the outcomes. Conducting subgroup analyses for these characteristics might not be feasible due to the limited number of studies available. This meta-analysis underscores the need for further high-quality randomized control trials that address the everyday clinical decisions faced by infertility specialists. Additionally, there is a call for comprehensive studies on the cost-effectiveness of such interventions.

## Conclusion

Our findings indicate that for infertile women, undergoing office hysteroscopy prior to artificial reproductive technology, despite previous imaging studies such as transvaginal sonography or hysterosalpingography showing no apparent intrauterine lesions, could enhance live birth and clinical pregnancy rates. Addressing lesions that imaging studies may have falsely missed—particularly endometrial polyps, submucosal fibroids, and endometritis—appears to positively influence ART outcomes. These lesions are known to adversely affect pregnancy rates, and thus, their identification and treatment through hysteroscopy should be considered.

Office hysteroscopy stands out for its safety, ease of use, diagnostic accuracy, and high patient tolerability. While current evidence indicates an increase in clinical pregnancy and live birth rates following outpatient hysteroscopy, prevailing guidelines and our perspective do not support routine use of outpatient hysteroscopy solely for enhancing live birth rates. The current evidence highlights a pressing need for further research to determine the value of hysteroscopy as a routine screening method for all women undergoing assisted reproductive technologies. Future studies should focus not only on the efficacy of hysteroscopy in improving fertility outcomes but also on its cost-effectiveness and impact on patient care pathways.

### Electronic supplementary material

Below is the link to the electronic supplementary material.

**Supplementary Material: Supplementary Figure S1.** Quality assessment using the Cochrane Risk of Bias tool for randomized controlled trials

**Supplementary Material: Supplementary Figure S2.** Forest plot comparing outcomes between the hysteroscopy and control groups using intention-to-treat approach

**Supplementary Material: Supplementary Figure S3.** Forest plots after sensitivity testing by excluding Elsetohy study comparing outcomes between the hysteroscopy and control groups

**Supplementary Material: Supplementary Table S1.** Characteristics of the infertility condition and artificial reproductive treatment of the included RCTs

**Supplementary Material: Supplementary Table S2.** Comparative analysis of data pre- and post-sensitivity testing by excluding Elsetohy study

## Data Availability

No datasets were generated or analysed during the current study.

## Abbreviations

ART: Artificial reproductive technology
CE: Chronic endometritis
CI: Confidence intervals
HSG: Hysterosalpingography
H&E: Hematoxylin and eosin
ICSI: Intracytoplasmic sperm injection
IHC: Immunohistochemistry
ITT: Intention-to-treat
IUI: Intrauterine insemination
IVF: In vitro fertilization
PP: Per-protocol
PRISMA: Preferred Reporting Items for Systematic Reviews and Meta-Analyses
RCT: Randomized controlled trials
RR: Risk ratios
TVS: Transvaginal sonography
