# Supplementary figures and images for: Outpatient hysteroscopy impact on subsequent assisted reproductive technology: a systematic review and meta-analysis in patients with normal transvaginal sonography or hysterosalpingography images
Source: Reprod Biol Endocrinol. 2024 Feb 1;22:18. doi: 10.1186/s12958-024-01191-0 (PMC10832084; doi:10.1186/s12958-024-01191-0)

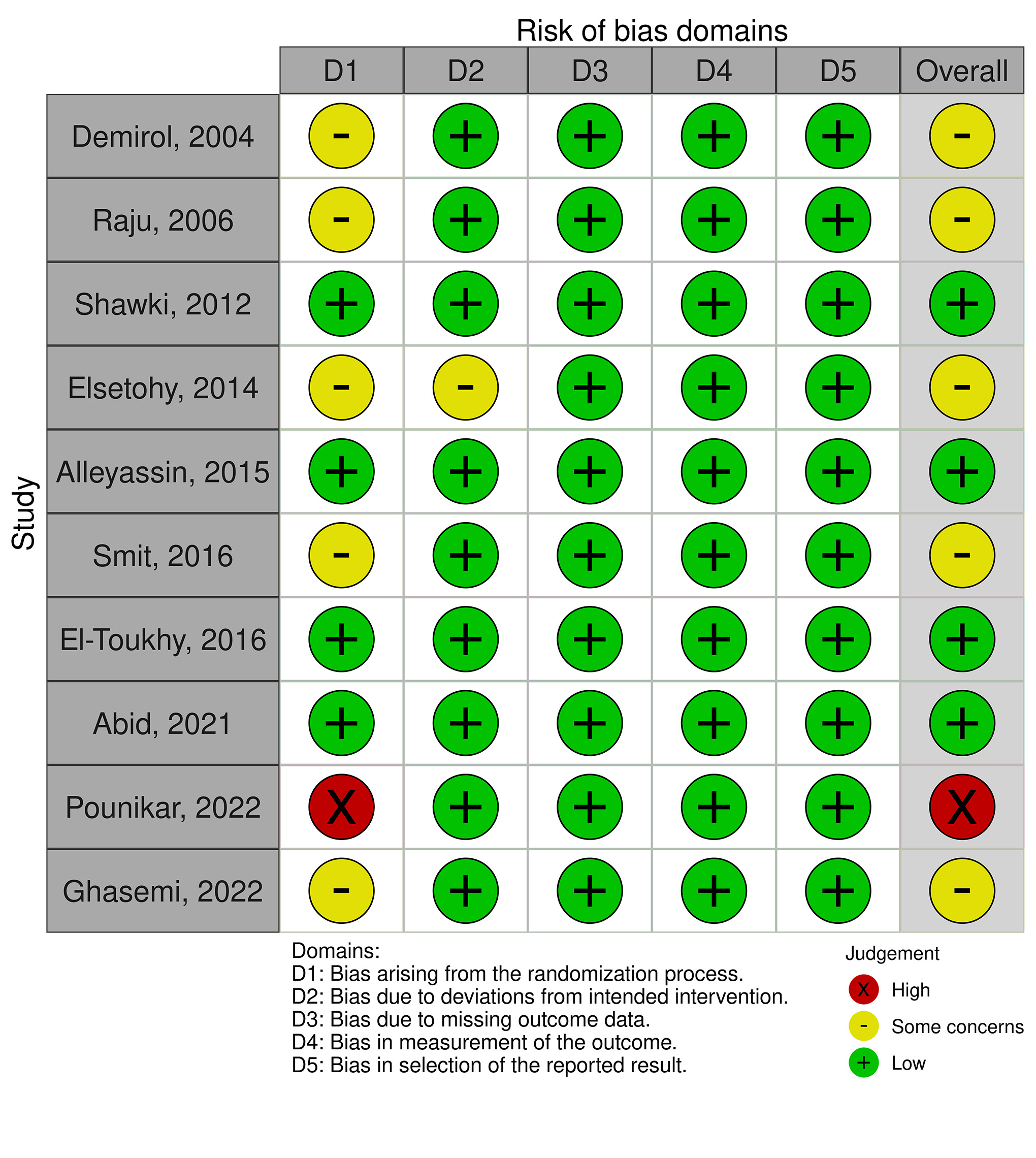

Supplement: Supplementary file 1 — Supplementary Material: Supplementary Figure S1. Quality assessment using the Cochrane Risk of Bias tool for randomized controlled trials [file 12958_2024_1191_MOESM2_ESM.jpg]

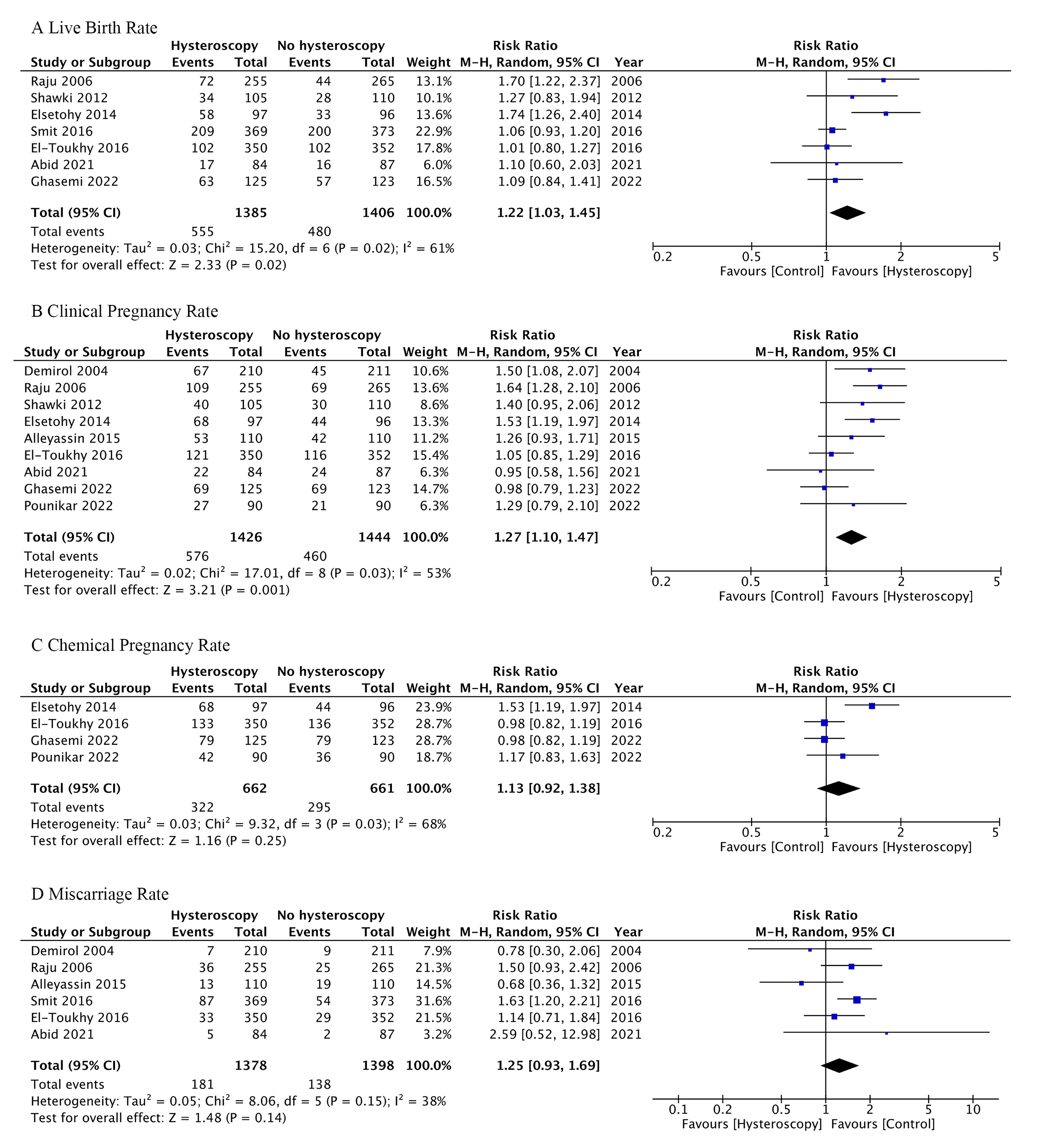

Supplement: Supplementary file 2 — Supplementary Material: Supplementary Figure S2. Forest plot comparing outcomes between the hysteroscopy and control groups using intention-to-treat approach [file 12958_2024_1191_MOESM3_ESM.jpg]

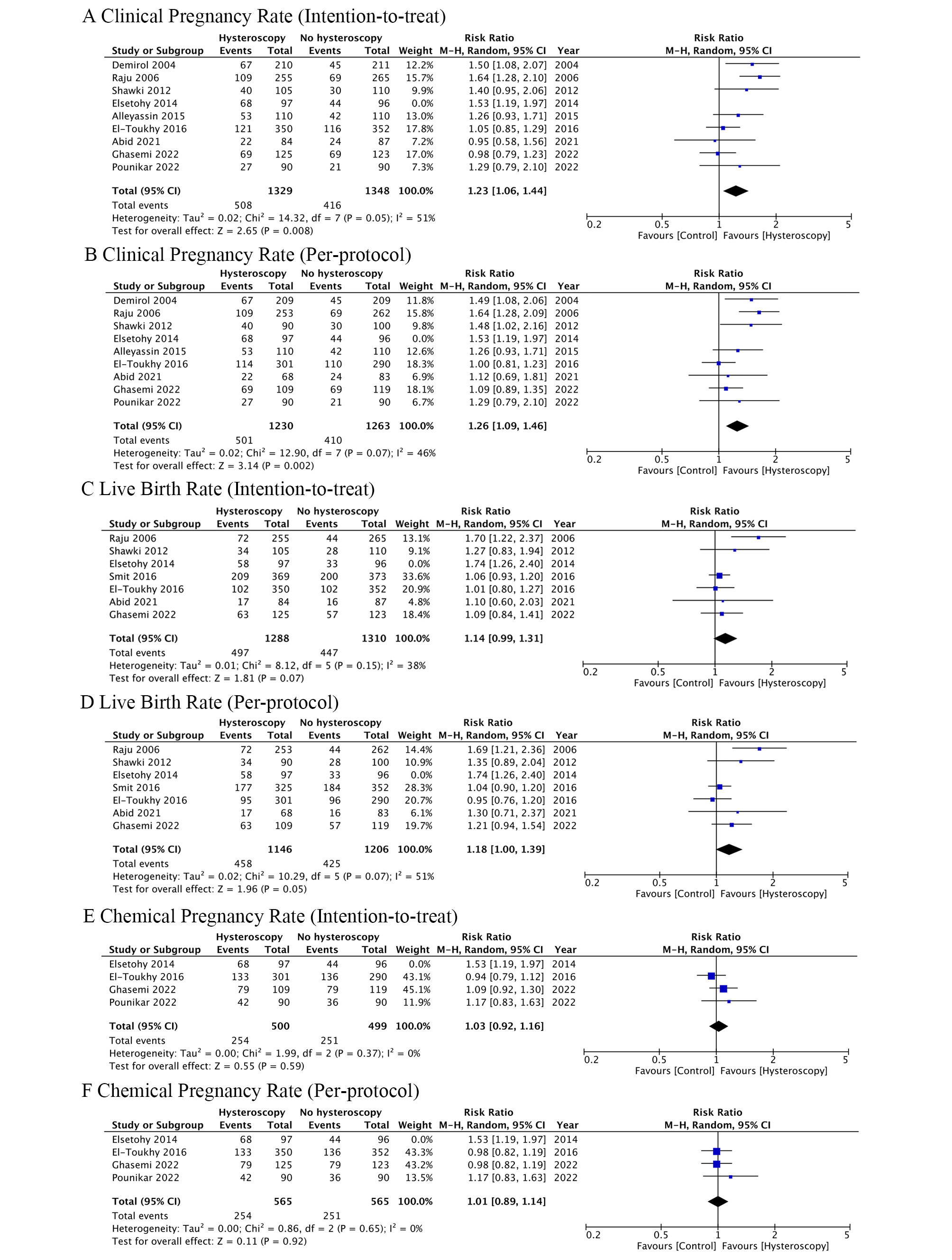

Supplement: Supplementary file 3 — Supplementary Material: Supplementary Figure S3. Forest plots after sensitivity testing by excluding Elsetohy study comparing outcomes between the hysteroscopy and control groups [file 12958_2024_1191_MOESM4_ESM.jpg]
