# Supplementary material for: Outpatient hysteroscopy impact on subsequent assisted reproductive technology: a systematic review and meta-analysis in patients with normal transvaginal sonography or hysterosalpingography images
Source: Reprod Biol Endocrinol. 2024 Feb 1;22:18. doi: 10.1186/s12958-024-01191-0 (PMC10832084; doi:10.1186/s12958-024-01191-0)
Supplement: Supplementary file 4 — Supplementary Material: Supplementary Table S1. Characteristics of the infertility condition and artificial reproductive treatment of the included RCTs [file 12958_2024_1191_MOESM1_ESM.docx]

**Supplementary Table S1 Characteristics of the infertility condition and artificial reproductive treatment of the included RCTs**

| Author, publication year | Mean age (y/o) | Infertility type | Infertility cause | Previous failed cycles (times) | Duration of infertility (mean years) | Fertilization | Embryo transfer |
| --- | --- | --- | --- | --- | --- | --- | --- |
| Demirol et. Al. (2004) | 32 | All primary infertility | Ovulatory 33.5%  Male 27%  Idiopathic 39.6% | 2.8 | 6.1 | All IVF | Fresh, on day 3 |
| Raju et. Al. (2006) | 27 | NA | Ovulatory 45.7%  Endometriosis 37.1%  Tubal 17.1%  Male 68.9%  Combine 20.5% | 2.6 | 7.0 | IVF 60.9%  ICSI 39.1% | Fresh, on day 3 |
| Shawki et. Al. (2012) | 32 | Primary 61.4%  Secondary 38.5% | Ovarian 26.8  Tubal 27.3  Male 22.8%  Unexplained 20.5%  REPL >8 times 2.3% | NA | 8.4 | All ICSI | Fresh |
| Elsetohy et. Al. (2014) | 31 | Primary 63.7%  Secondary 36.3% | Ovarian 16.6%  Tubal 26.9%  Male 51.3%  Unexplained 20.7% | NA | 5.8 | All ICSI | Fresh |
| Alleyassin et. Al. (2015) | 29 | NA | Ovarian 31.05%  Tubal/peritoneal 21.8%  Male 31.4%  Unexplained 15.8% | NA | 4.7 | All ICSI | Fresh, on day 3 |
| Smit et. Al. (2016) | 33 | Primary 65%  Secondary 35% | Ovulation 14%  Endometriosis 4.5%  Tubal 10.0%  Male 54.5%  Unexplained 29.0% | NA | 2.3 | IVF or ICSI | Fresh 67%, frozen 18.0% |
| El-Toukhy et. al. (2016) | 33 | Primary 89.9%  Secondary 10.1% | Ovulation 6.5%  Endometriosis 9.0%  Tubal 16.0%  Male 45%  Unexplained 16.5%  Combined 6.5% | 1.0 | 4 | IVF 22%  ICSI 78% | Fresh 55%, frozen 45% |
| Abid et. al. (2021) | 32 | Primary 89.5%  Secondary 10.5% | Tubal 5.3%  Male 84.8%  Mutifactorial 6.4%  Unexplained 3.5% | NA | 4.5 | NA | Fresh, on day 2 or 3 |
| Pounikar et. al. (2022) | 33 | Primary or secondary | NA | 1 time: 24%  >1 times: 66% | 7.9 | NA | NA |
| Ghasemi et. Al. (2022) | 30 | All primary | NA | NA | NA | IVF or ICSI | All fresh ET on day 3 in the beginning, 40% underwent second frozen ET |

**ET**, embryo transfer; **NA**, not available; **IVF**, in vitro fertilization; **ICSI**, intracytoplasmic sperm injection; **REPL**, repeated early pregnancy loss
