# Supplementary material for: Outpatient hysteroscopy impact on subsequent assisted reproductive technology: a systematic review and meta-analysis in patients with normal transvaginal sonography or hysterosalpingography images
Source: Reprod Biol Endocrinol. 2024 Feb 1;22:18. doi: 10.1186/s12958-024-01191-0 (PMC10832084; doi:10.1186/s12958-024-01191-0)
Supplement: Supplementary file 5 — Supplementary Material: Supplementary Table S2. Comparative analysis of data pre- and post-sensitivity testing by excluding Elsetohy study [file 12958_2024_1191_MOESM5_ESM.docx]

| **Supplementary Table S2 Comparative analysis of data pre- and post-sensitivity testing by excluding Elsetohy study** | | | | | | |
| --- | --- | --- | --- | --- | --- | --- |
|  | Before excluding | | | After excluding | | |
|  | RR | CI | I^2^ | RR | CI | I^2^ |
| Clinical pregnancy rate (ITT) | 1.27 | 1.10-1.47 | 53% | 1.23 | 1.06-1.44 | 55% |
| Clinical Pregnancy rate (PP) | 1.29 | 1.13-1.48 | 47% | 1.26 | 1.09-1.46 | 46% |
| Live birth rate (ITT) | 1.22 | 1.03-1.45 | 61% | 1.14 | 0.99-1.31 | 38% |
| Live birth rate (PP) | 1.26 | 1.05-1.50 | 65% | 1.18 | 1.00-1.39 | 51% |
| Chemical pregnancy rate (ITT) | 1.13 | 0.92-1.38 | 68% | 1.01 | 0.89-1.14 | 0% |
| Chemical pregnancy rate (PP) | 1.15 | 0.94-1.4 | 69% | 1.03 | 0.92-1.16 | 0% |
| In the Elsetohy 2014 study, the live birth rate following artificial reproductive technology was 64.4%, higher than the global average. Therefore, we excluded it in our sensitivity analysis. After excluding the Elsetohy study, the clinical pregnancy rate was significantly higher in the hysteroscopy group. There was a trend towards increased live birth rates with hysteroscopy, though not statistically significant. Heterogeneity in our analysis was reduced in both the intention-to-treat (from 61% to 38%) and per-protocol approaches (from 65% to 51%).  The Elsetohy paper is noteworthy for its rigorous peer review and inclusion in two major meta-analyses, offering insights into diagnostic hysteroscopy across various patient groups. While its exclusion might reduce heterogeneity, it could potentially impact the objectivity of our findings. Our discussion, therefore, takes a balanced approach, considering insights from other research and guidelines. We emphasize the importance of cost-effectiveness given the moderate benefits, instead of advocating routine outpatient hysteroscopy solely for increasing the live birth rate.  In line with the reviewer's suggestion, we have chosen to include these results in a supplementary table, thereby maintaining the integrity of the main results in the manuscript. | | | | | | |
|  | | | | | | |
